# 91348 A mixed methods analysis of hurdles to productivity among T and K awardees

**DOI:** 10.1017/cts.2021.577

**Published:** 2021-03-30

**Authors:** Margaret Schneider, Lisa Jones, Amanda Woodward

**Affiliations:** University of California, Irvine

## Abstract

ABSTRACT IMPACT: Recommendations for increasing trainee productivity will be highlighted. OBJECTIVES/GOALS: Using a combination of qualitative (interview) and quantitative (publications tracking) data, we undertook to describe the hurdles and concerns impeding academic accomplishments among T and K awardees at one CTSA hub and to examine whether hurdles at 6 months would predict academic output within one year following completion of the training program. METHODS/STUDY POPULATION: Semi-structured interviews were conducted with 29 trainees (28 TL1 and 8 KL2) 6 months into their training. Interview transcripts were analyzed using Atlas.ti to identify hurdles (factors that had already impeded research progress) and concerns (future challenges anticipated by the trainee). PubMed searches yielded the number of publications within one year of exiting the training program. Frequencies of hurdles and concerns were examined to characterize the factors most likely to impact trainee progress during the first 6 months of their training program. Among 18 trainees who had completed their training, the mean number of publications within one year of exiting the program (identified via verified PubMed searches) was compared across the total number of hurdles reported at 6 months (range = 0 to 3). RESULTS/ANTICIPATED RESULTS: The thematic analysis yielded 19 categories of hurdles and 14 categories of concerns. The top three hurdles were technological challenges (e.g., issues with equipment or data reduction; reported by 63% of trainees), professional competing responsibilities (40%), and navigating collaborations (30%). The top three concerns were future funding (33%), potential as an independent researcher (27%), and institutional context (e.g., departmental structure; 23%). The number of hurdles reported at 6 months significantly predicted number of publications one year post-exit (F (3,14) = 3.14, p < .05). Trainees reporting zero hurdles generated a mean of 8.67 publications; those with 3 hurdles generated a mean of 2.4 publications. DISCUSSION/SIGNIFICANCE OF FINDINGS: Future concerns were completely different from past hurdles, suggesting that the issues impeding research progress are not anticipated. Results suggest trainees would benefit from training related to how to balance competing professional responsibilities and navigate collaborations and that early attention to hurdles may enhance productivity.

